# Glia in amyotrophic lateral sclerosis and spinal cord injury: common therapeutic targets

**DOI:** 10.3325/cmj.2019.60.109

**Published:** 2019-04

**Authors:** Jelena Ban, Cynthia Sámano, Miranda Mladinic, Ivana Munitic

**Affiliations:** 1 Laboratory for Molecular Neurobiology, Department of Biotechnology, University of Rijeka, Rijeka, Croatia; 2 Laboratorio de Biología Celular, Departamento de Ciencias Naturales, Universidad Autónoma Metropolitana, Unidad Cuajimalpa, Mexico City, Mexico; 3 Laboratory for Molecular Immunology, Department of Biotechnology, University of Rijeka, Rijeka, Croatia

## Abstract

The toolkit for repairing damaged neurons in amyotrophic lateral sclerosis (ALS) and spinal cord injury (SCI) is extremely limited. Here, we reviewed the *in vitro* and *in vivo* studies and clinical trials on nonneuronal cells in the neurodegenerative processes common to both these conditions. Special focus was directed to microglia and astrocytes, because their activation and proliferation, also known as neuroinflammation, is a key driver of neurodegeneration. Neuroinflammation is a multifaceted process that evolves during the disease course, and can be either beneficial or toxic to neurons. Given the fundamental regulatory functions of glia, pathogenic mechanisms in neuroinflammation represent promising therapeutic targets. We also discussed neuroprotective, immunosuppressive, and stem-cell based approaches applicable to both ALS and SCI.

The nervous system was traditionally investigated by focusing primarily on neurons as main functional units (“neurocentric” view) (1). Recently, this standpoint has changed, and glial cells are now recognized as active participants in virtually all functions of the nervous system. Neurons, which could arguably be considered the least self-sufficient cells in the body, need glia for development, function, maintenance, and plasticity (2-6). However, in central nervous system (CNS) insults the key homeostatic functions of glia may be compromised, turning them into major drivers of neuronal death. In this review, we focused on the role of astrocytes and microglia in two devastating diseases of the nervous system without effective therapies, amyotrophic lateral sclerosis (ALS) and spinal cord injury (SCI). Due to their distinct etiology, these diseases are rarely discussed together. However, they share several key pathogenic mechanisms, thus allowing us to pinpoint potential common targets for therapeutic intervention.

## Glial cell subsets and functions

Glial cells of the central nervous system (CNS) comprise astrocytes, oligodendrocytes, microglia, and ependymal cells. The shortest description of CNS glia could be “homeostasis-maintaining cells” (5,7). Except for microglia, they all originate from radial glia, neural stem cells (NSC)/neural progenitor cells (NPC) present in large numbers only during the embryonic development (7,8). Due to limited presence of NSC, neurogenesis in adults is restricted to the so-called neurogenic niches (subventricular zone and the subgranular layer of the hippocampus) (9,10). Ependymal cells, primarily responsible for production and regulation of cerebrospinal fluid (CSF), retain stem cells properties (11). Here, we primarily focused on two cell types with key homeostatic roles: astrocytes and microglia.

Astrocytes perform multiple neuroprotective functions. Due to the impressive number of receptors, channels, and transporters, they regulate the exchange of water, ions, neurotransmitters, and various metabolites. They also have a fundamental structural and functional role in maintaining the brain-blood barrier (BBB) and its spinal cord equivalent, the blood-spinal cord barrier (BSCB), and release the neurotrophic factors such as brain-derived neurotrophic factor (BDNF), nerve growth factor (NGF), glial-derived neurotrophic factor (GDNF), and vascular endothelial growth factor (VEGF) (3,7). Other important functions include synaptic formation, maturation, pruning, transmission, and plasticity. Although astrocytes were historically attributed a merely passive role in the synaptic activity, due to their proximity and bidirectional communication with neurons, a new concept of the tripartite synapse has been proposed, consisting of glia and presynaptic and postsynaptic neurons (12). Finally, in response to damage, astrocytes take part in brain protection (reactive astrogliosis, scar formation, secretion of proinflammatory factors). These immune functions of astrocytes intricately depend on microglia as the primary damage sensors, demonstrating a tight interaction between the glial subsets (13).

Microglia are the only resident innate immune cells in the CNS parenchyma. In response to tissue damage and/or pathogens, they trigger inflammatory responses similarly to peripheral macrophages (14), although these responses are less robust. This is partly because their precursors enter the CNS during the early embryonic development and are normally not replaced by infiltrating monocytes (15,16). Similar to astrocytes, microglia also contribute to synaptic pruning and the secretion of neurotrophic factors including BDNF, GDNF, and insulin-like growth factor-1 (IGF-1). Their activation state closely mirrors their microenvironment: if the surrounding neurons are not under stress, they signal microglia to remain quiescent by expressing negative costimulatory molecules CD200 and chemokine CX3CL1 (fractalkine) (17). However, various danger signals derived from damaged or dying cells, including ATP, protein aggregates, and/or loss of CD200- and CX3CL1-signaling, activate microglial inflammatory responses (18,19). The activation of microglia, known as microgliosis, is accompanied by proliferation and secretion of numerous proinflammatory cytokines (TNF, IL-1β, etc) and chemokines, generation of reactive oxygen and nitrogen species (ROS and RNS, respectively), and phagocytosis of damaged tissues. As mentioned above, microglial activation also orchestrates the activation of astrocytes (13). If the primary damage resulted in the breakdown of the BBB or BSCB, microglia temporarily patch up the barrier and diminish the infiltration of peripheral cells. In contrast, if they cannot contain the damage, they actively recruit immune subsets to the damage site by secretion of various chemokines.

There are still many open questions on glial subsets and functions, including the mystery of their exact number (the estimated number of glial cells has recently decreased 10-fold to reach 1:1 ratio to neurons) and the heterogeneity of individual subsets (exclusive subtype-specific markers are still missing) (7,20). However, as evident from the abovementioned functions, astrocytes and microglia cooperate during development and adult life to regulate synaptic functions and provide trophic support, and most importantly, to trigger neurorepair following injury (21). Neurorepair begins upon elimination of damaged tissues when the proinflammatory response of microglia and astrocytes subsides, and an anti-inflammatory response starts to predominate. The latter is marked by the secretion of anti-inflammatory cytokines, such as IL-4 and IL-10. However, the extensive and/or prolonged damage can preclude efficient repair, resulting in highly damaging chronic neuroinflammation, as is the case in ALS and SCI.

## The role of glia in amyotrophic lateral sclerosis

ALS is the most common adult motor neuron disease and the fastest progressing neurodegenerative disease (22,23). It is marked by an unusual heterogeneity at several levels: 1) it can be caused by mutations in >30 unrelated genes; nonetheless, the majority of cases are sporadic with unknown underlying genetic and environmental component; 2) the rate of progression, onset site, and initial ratio of upper/lower motor neuron involvement differ substantially in both familial and sporadic cases; 3) the loss of motor neurons spreads to adjacent regions until practically all motor neurons are affected; 4) death occurs within 2-5 years upon diagnosis, although in rare cases, disease lasts 10 years or more (24,25). The death of the motor neurons in the spinal cord, brainstem, and cerebral cortex, is a rare common denominator within the complexity of ALS (23). However, it is still unclear how mutations in different genes, most common of which encode for chromosome 9 open reading frame 72 (C9ORF72), superoxide dismutase 1 (SOD1), TAR DNA-binding protein 43 (TDP-43), Fused in Sarcoma (FUS) and TANK-binding kinase 1 (TBK1), cause neuronal death (25,26). The affected neurons in >95% of ALS cases, regardless of genetic background, contain TDP-43 aggregates, which spread to the neighboring neurons (27). SOD1 and other aggregation-prone proteins act similarly in specific mutation carriers, but we do not know the exact reason why the aggregates are toxic to neurons and if they are the earliest detected pathology. However, they stimulate the activation of microglia and astrocytes, thus making glia essential for the neurodegenerative process (28-30).

The decisive role of glia in ALS has been mapped in elegant conditional genetic models, which were based on the transgenic mouse model containing an aggregate-prone patient SOD1 mutation (mSOD1) and exhibiting early onset hind limb paralysis and premature death (31). Remarkably, if the mutated SOD1 transgene expression is restricted to neurons, ALS does not develop, suggesting that neurons do not die if the surrounding glia are healthy (32,33). When the mSOD1 transgene was conditionally deleted in individual glial subsets (microglia, astrocytes or oligodendrocytes), the ALS progression was substantially slowed down (34-36). In contrast, when the innate immunity was chronically stimulated by systemic lipopolysaccharide (LPS), ALS symptoms in mice exacerbated (37). mSOD1 carrying microglia and astrocytes from animal models and familial and sporadic ALS patients are able to kill motor neurons (but not interneurons) both i*n vivo* and *in vitro* (38-40). Finally, given the variability in ALS onset time and site, limited penetrance of many ALS mutations, and the substantial differences in disease progression that are present even in familial ALS cases, it is not too ambitious to hypothesize that glial cells are the key determinants of disease onset and/or progression. If this is the case, neuronal damage could be prevented or delayed by improving the homeostatic functions of glia and/or suppressing the neurotoxic inflammation.

## The role of glia in spinal cord injury

SCI, of traumatic or non-traumatic origin, is a devastating condition with high incidence, causing mortality or severe neurological deficits and permanent disability (41,42). Even though considerable progress has been made in understanding molecular pathways and cellular changes involved in the pathophysiology of SCI, no current therapies are able to restore neuronal connections and re-establish neuronal circuits responsible for complex functions such as standing or walking. Since a hallmark of SCI are neuronal death and deficits, the research has been mostly focused on axonal regeneration, neuronal plasticity, and neuroprotective drugs able to prevent neuronal death in secondary injury responses. The pathophysiology of SCI involves active participation of numerous glial cells (astrocytes, microglia, oligodendrocytes, pericytes, etc), which can both facilitate repair or potentiate damage. Potential therapies could thus target glial cells, their mutual interactions, or interactions with neurons (43,44).

## Common pathophysiologic mechanisms in amyotrophic lateral sclerosis and spinal cord injury

Microglia in ALS act as a double-edged sword by exerting 1) neuroprotective effect in the early stages by limiting the damage via phagocytosis of dead neurons and protein aggregates, and secreting anti-inflammatory and neurotrophic factors, and 2) neurotoxic effect in the later phases by activating astrocytes. The astrocyte activation leads to a positive-feedback loop, in which homeostatic functions of both cell types fail and hyperinflammation, dominated by TNF, IL-1β, IL-6 cytokines and oxidative damage, spins out of control and causes collateral neuronal damage (13,45). Of note, in a proinflammatory environment, excitotoxic neuron death is increased because TNF enhances glutamatergic transmission (46). To the affected CNS areas, microglia also attract T cells (47,48), whose effect can also be either beneficial or toxic. The early phases are characterized by the predominance of the regulatory T cells (Tregs), which support the anti-inflammatory microglia, whereas the later stages (or the fast progressing disease course) are characterized by their diminishing number and the predominance of effector T cells (49). Therefore, the proinflammatory and anti-inflammatory milieu at the affected sites determines the speed of disease progression and presents a tempting therapeutic target.

SCI pathophysiology, similar to that of ALS, is complex and includes multifold events that extend over time and space. In traumatic SCI, the initial traumatic insult that mechanically damages spinal cells and blood vessels at the injury site is succeeded by a secondary injury cascade. This cascade consists of inflammation, edema, hemorrhage, ischemia, and excitotoxicity, which induce ionic disbalance and the death of neuronal and glial cells (by necrotic, apoptotic, and other programmed death pathways), causing demyelination, further inflammatory cell infiltration, astrogliosis, and the reorganization of vasculature, extracellular matrix, and neuronal circuits (41,42). The subsequent formation of a cyst, surrounded by a fibrous scar (containing astrocytes, pericytes, and ependymal cells), impedes axonal regrowth and regeneration. These secondary events effuse the damage of spinal tissue significantly outside the epicenter of injury and represent a source of multiple attractive therapeutic targets. On the other hand, the fibrotic scar has a beneficial, tissue-preserving role, confining inflammation to the lesion epicenter and restricting tissue damage and neural loss after SCI, mostly by neural stem cell-derived scar component (44,50). Similar to astrogliosis in ALS, neuroprotective scar formation is contingent on microglial activation (51). Microglial activation also limits the damage by mitigating the recruitment of peripheral macrophages and leukocytes. However, if the damage is too extensive, microglia contribute to the pathology. Therefore, the activated microglia have both beneficial and detrimental effects on the spinal tissue after injury, influencing multiple factors that perform a variety of roles, from promotion of neuronal damage to neuroprotection and promotion of axonal growth (44).

The physical disruption of capillaries and BSCB breakdown in SCI present an acute threat and cause rapid infiltration of blood-borne factors and peripheral blood cells, such as monocytes and neutrophils (52). The initial acute insult and cell infiltration are replaced with increased permeability linked to revascularization of the scar tissue (53,54). In contrast to SCI, animal ALS models have clearly demonstrated minor infiltration of peripheral blood cells and increase in the microglial number strictly due to local proliferation (15). However, various subtle chronic defects in BSCB have been reported in both animal models and patient tissues, including increased endothelial permeability, decrease in tight junction proteins, microhemorrhages, and antibody deposition (55-57). Of note, reactive astrocytes and microglia in both in ALS and SCI release proinflammatory factors, ROS, glutamate, matrix metalloproteinases (MMPs), and VEGF, which down-regulates the expression of tight junction proteins. Therefore, BSCB is compromised in both SCI and ALS, but with different kinetics and magnitude, and should thus be targeted by therapies customized to the disease stage.

## Common therapeutic approaches in amyotrophic lateral sclerosis and spinal cord injury

### Neuroprotective agents

Since in both SCI and ALS, CNS responds to injury by employing similar pathological pathways and cell-death mechanisms, the therapeutic targets in both diseases might be similar. Riluzole, the only widely used drug for ALS, prolongs the life of ALS patients for 2-3 months (58). Despite its complex and incompletely defined mechanism of action, it exerts several effects beneficial for ALS and SCI: 1) decrease of presynaptic glutamate release, 2) reduction of the persistent Na^+^ current, 3) facilitation of glutamate uptake, and 4) inhibition of neuronal excitability (59-62). It has recently been tested in numerous animal models of spinal cord ischemic and traumatic injury, where it exerted neuroprotective effects on spinal gray matter and neuromodulation (63-65). In two clinical trials it improved the motor scores and provided other benefits for SCI patients, while its efficacy is currently under extensive clinical investigation (41,66).

A number of neurodegenerative disease models, including those of ALS, have shown neuroprotective function of arimoclomol, leading to clinical testing of its therapeutic potential (67,68). Arimoclomol is a coinducer of heat shock proteins (HSP), molecular chaperones involved in heat shock response, a major defense mechanism against stress or injury (69). Similarly, natural compound celastrol, which induces HSP, has been tested as a neuroprotectant in different animal and *in vitro* models of neurodegeneration, including ALS and SCI, with a beneficial outcome (69,70). Other mechanisms of action of celastrol could be relevant for both ALS and SCI, including its anti-inflammatory role (71). Furthermore, since neuronal tolerance to stress is not entirely dependent on their own HSP, it should be elucidated if the effect of both arimoclomol and celastrol on adjacent glial cells can supplement neuronal HSP after SCI.

A free radical scavenger edaravone has recently been approved for ALS in Japan and USA (2015 and 2017, respectively) as a riluzole add-on therapy because it slightly slowed down disease progression in patients in the early disease stages (72,73). Edaravone has previously been approved for acute-phase cerebral infarction, but its efficacy in SCI has not been tested (74).

Although various neurotrophic factors (GDNF, BDNF, IGF-1, etc) promote neuronal survival and regeneration, they have not lead to clinical improvement in SCI and ALS (41,72), presumably because single factors were insufficient to exert therapeutic effects and/or because of the complexity of trophic factor signaling. Overall, since direct neuroprotective agents provide only limited or no effects, and since glia exhibit superior plasticity to neurons, a large number of experimental therapies that directly target glia are currently studied.

### Immunosuppressive and anti-inflammatory approaches

Given that chronic neuroinflammation is toxic to neurons, a large number of preclinical and clinical trials attempted immunosuppressive and/or anti-inflammatory approaches (72). Contrary to predictions, general anti-inflammatory drugs proved to be rather inefficient in ALS. For example, late-stage clinical trials showed the ineffectiveness of anti-inflammatory COX-2 inhibitors and minocycline, whereas small-scale ALS studies showed the ineffectiveness of immunosuppressive glucocorticoid methylprednisolone (72,75). CNS-targeted glucocorticoid reduced astrogliosis and neuronal loss in cranial motor nuclei but failed to preserve lower motor neurons or improve motoric and behavioral symptoms in mSOD1 mice (76). However, methylprednisolone has for a long time been widely accepted as a standard of care in SCI without being officially approved by Food and Drug Administration (41). In experimental animal models it beneficially affected the white matter oligodendrocytes and astrocytes, but with questionable functional recovery (77-80). Methylprednisolone is less used today because of its moderate efficacy and recognized side effects (66).

The nuclear factor kappa-light-chain-enhancer of activated B cells (NF-κB) is the master transcriptional factor for microglial and astrocyte inflammatory responses. It is activated in various CNS pathologies, including ALS and SCI, and its therapeutic potential has been demonstrated by various preclinical studies (81-83). For example, NF-κB inactivation in astroglia reduced the production of chondroitin sulfate proteoglycans and proinflammatory cytokines and chemokines, thus promoting oligodendrogenesis, white matter preservation, and functional recovery after traumatic SCI (84,85). Drugs that target NF-κB *in vivo* are still under research, however mouse ALS model expressing human TDP-43 mutation showed that the extract of herbal medicine *Withania somnifera* (Ashwagandha) with anti-inflammatory properties reduced NF-κB activity, neuroinflammation, TDP-43 aggregation, and improved neuromuscular innervation (86). Another inflammatory signaling pathway leading to production of the immunomodulatory cytokine IFN-β, dependent on TBK1 and optineurin, has recently been found to be disrupted in ALS patients and mouse models (87,88). As IFN-β can suppress proinflammatory cytokine production and exert neuroprotective effect in multiple sclerosis (89), it remains to be investigated whether it can also have a protective effect in ALS and SCI.

Genetic deletions of major proinflammatory cytokines, such as IL-1β and TNF, did not reduce disease progression in mSOD1 ALS mice models (90,91). This suggests the inefficiency of targeting single proinflammatory factors. In contrast, anti-inflammatory IL-10 cytokine blockade in mSOD1 ALS models activated microglia and precipitated the disease. On the contrary, IL-10 delivery via viral vectors substantially slowed down ALS progression (92). IL-10 reduced several secondary effects in animal models of SCI by facilitating functional recovery (93,94). The positive effects of IL-10 can be potentiated by using Schwann cell and olfactory cell grafts, and drugs such as methylprednisolone, minocycline, hyperbaric oxygen, etc. Overall, IL-10 shows an excellent promise for treating SCI, however its potential secondary immunosuppressive effects during chronic application (pneumonia, peripheral neuropathy, etc) need to be addressed (95).

Rat mSOD1 models have recently demonstrated that ALS progression was reduced by tyrosine kinase inhibitor masitinib (96,97). It was shown to inhibit signaling pathways in several innate immune cells including microglia, mast cells, and neutrophils, however clinical trials are still undergoing (72). Since masitinib targets several key inflammatory cells and pathways, it could be an interesting treatment target in SCI.

### Cell-based approaches

Cell transplantation is an attractive approach in both ALS and SCI, but despite almost 20 years of research it is still not a reliable clinical option (98,99). Two major goals of cell transplantation are 1) to provide neuroprotection by limiting tissue damage, which is more feasible, and 2) to support neuroregeneration by cell replacement, remyelination, and repair of endogenous circuits in the spinal cord, which remains challenging. Progenitor cell types most commonly used in experimental ALS therapies include NSCs obtained from fetal CNS or differentiated from adult tissues (for example, iPSC-derived glial-rich progenitors [GRPs]), mesenchymal stem cells (MSCs), and hematopoietic stem cells (HSCs) (98,100-102). HSCs showed promising results in preclinical studies, and transplantation of healthy bone marrow into mSOD1 transgenic mice diminished motor neuron loss and prolonged survival. However, in clinical trials HSC transplantation upon total body irradiation was unsuccessful (103,104). GRP, NSC, and MSC approaches have been more successful, with the best cell yields and survival reported upon multifocal intralesional transplantation. Transplanted GRPs were shown in an mSOD1 ALS mouse model to generate astrocytes, reduce microgliosis, decrease motor neuron loss, and mitigate disease progression (105). Since glutamate transporter GLT-1-deficient GRPs were ineffective, these effects could at least in part be ascribed to decreased excitotoxicity. Undifferentiated multipotent NSC have also been proven to be safe and considerably effective upon intralesional application in mSOD1 ALS models: they preserved motor neurons and neuromuscular junctions, diminished microgliosis and astrogliosis, and increased the secretion of neurotrophic factors (106). They were effective although mere ~ 1% of transplanted cells differentiated into neurons and ~ 10% to astrocytes or oligodendrocytes, suggesting that the protective effect was largely mediated by undifferentiated NSCs. Interestingly, these mechanisms of action make NSCs similar to MSCs. Indeed, the main neuroprotective effects of MSCs are supporting anti-inflammatory and immunomodulatory cytokine and trophic factor production and diminishing excitotoxicity (101,107). A major advantage of MSCs is their non-immunogenicity, which obviates the need for immunosuppression. Successful application of MSCs and NSCs in animal ALS models paved the way for the first clinical trials (108-110). Their safety was further confirmed in larger follow-up studies, which showed some preliminary evidence of efficacy (111-113). Although considerable progress has been made in stem cell-based therapies in ALS, the exact mechanisms of action, cell take/rejection, preparation, appropriate dose, and route of administration need to be better addressed before the efficacy is tested in large-scale studies.

Numerous cell types have also been considered for transplantation in SCI, including MSCs, NSCs, NPCs, oligodendrocyte progenitor cells (OPCs), iPSCs, as well as non-stem cells such as Schwann cells and olfactory ensheathing glial cells (OEG) (41,114). Commonly proposed mechanisms of action of transplanted cells in SCI include immunomodulatory and anti-inflammatory cytokine production, neuroprotection, axon sprouting/regeneration, myelin regeneration, and neuronal relay formation (115). Schwann cells transplanted to a rodent SCI model stimulated axonal regeneration, thus improving locomotor coordination (115,116). A similar procedure has been proven to be safe in a clinical trial with a 2-year follow-up in chronic SCI patients, providing preliminary evidence of partial recovery of sensorimotor activity (117). The OEG-based transplantation after experimental SCI had substantial overall efficacy (118), but few chronic SCI patients in clinical trials phase I/IIa functionally recovered (119). Because of the neuroprotective effects of MSCs and NSCs, their clinical utility has been extensively tested in chronic SCI (41). NSCs grafts attenuate reactive gliosis, and a fraction of them that differentiates in astrocytes participates in BSCB formation and extensively migrates out of the grafts, which is why they have been proposed as candidates for treating rodent SCI (120). In conclusion, similar to ALS, cell-based approaches in SCI have provided encouraging preclinical and clinical results, but we need to understand them better if we want to personalize and enhance their therapeutic efficacy.

## The role of the blood-brain and blood-spinal cord barrier in therapies

Crossing the BBB/BSCB is the biggest challenge for almost every CNS therapy. The efficiency of drug delivery could be increased by physical or chemical opening of the BBB by nanotechnological approaches that use nanocarriers and/or non-invasive intranasal drug delivery (121,122).

## Conclusion

**Figure 1:**
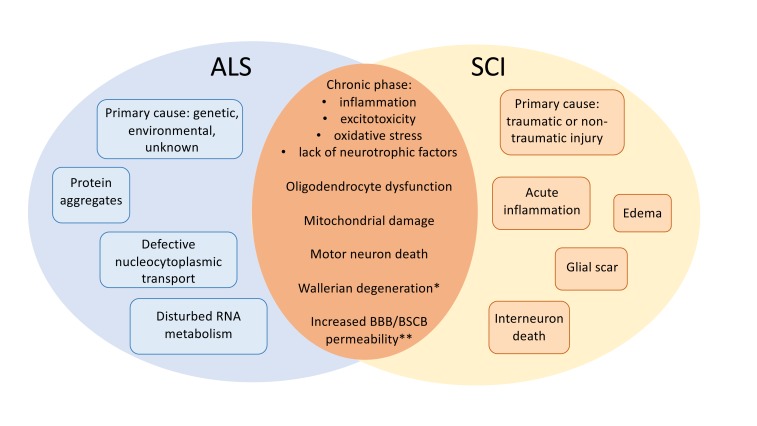
Amyotrophic lateral sclerosis (ALS) and spinal cord injury (SCI) hallmarks. Specific and common features of these neurodegenerative diseases. Asterisk: Wallerian degeneration, a typical dying-forward neurodegenerative process in SCI, has also been reported in ALS, although dying-back hypothesis is now gaining more ground (123); Double asterisk: the massive infiltration of peripheral blood cells is specific for SCI, whereas increased permeability to blood-borne factors is common to both ALS and SCI. BBB/BSCB – blood-brain/blood-spinal cord barrier.

Whereas ALS and SCI differ in their primary cause, what they have common are distal pathogenic mechanisms, most of which affect neuroinflammatory pathways of microglia and astrocytes (Figure 1). This opens the possibility of using common targets for therapeutic intervention. Here, we propose that the drugs shown to be effective in ALS, such as arimoclomol, masitinib, immunomodulatory cytokines, cell therapies, and others, should be evaluated as therapeutic candidates for SCI (and vice versa). Targeted therapies that would support protective glial functions while blocking their toxic function are still an elusive goal, but will likely offer wider possibilities than neuron-targeting therapies.
